# Ostomy patients’ perception of the health care received

**DOI:** 10.1590/1518-8345.2059.2961

**Published:** 2017-12-11

**Authors:** Candela Bonill-de las Nieves, Concepción Capilla Díaz, Miriam Celdrán-Mañas, José Miguel Morales-Asencio, Sandra Milena Hernández-Zambrano, César Hueso-Montoro

**Affiliations:** 1 Doctoral, RN, Servicio Andaluz de Salud, Almería, España.; 2 Graduate, Nursing, Antropologist, PhD, Professor, Facultad de Ciencias de la Salud. Universidad de Granada (Campus Ceuta), Ceuta, Spain; 3 Master’s, RN, Servicio Andaluz de Salud, Almería, España.; 4 PhD, Professor, Facultad de Ciencias de la Salud, Universidad de Málaga, Málaga, España.; 5 PhD, Professor and Researcher. Fundación Universitaria en Ciencias de la Salud, Bogotá, Colombia.; 6 PhD, Professor, Facultad de Ciencias de la Salud, Universidad de Granada, Granada, España.

**Keywords:** Colostomy, Ileostomy, Qualitative Research, Health Services, Patient Satisfaction, Health Personnel

## Abstract

**Aim:**

to describe ostomy patient’s perception about health care received, as well as their needs and suggestions for healthcare system improvement.

**Method:**

qualitative phenomenological study was conducted, involving individual and semi-structured interviews on the life experiences of 21 adults who had a digestive stoma. Participants were selected following a purposive sampling approach. The analysis was based on the constant comparison of the data, the progressive incorporation of subjects and triangulation among researchers and stoma therapy nurses. The software Atlas.ti was used.

**Results:**

perception of health care received is closely related to the information process, as well as training for caring the stoma from peristomal skin to diet. It is worthy to point out the work performed by stoma care nurses ensuring support during all stages of the process.

**Conclusion:**

findings contribute to address the main patients’ needs (better prepared nurses, shorter waiting lists, information about sexual relation, inclusion of family members all along the process) and recommendations for improving health care to facilitate their adaptation to a new status of having a digestive stoma.

## Introduction

The effects caused by a gastro-intestinal (GIT) stoma do not just exert physical and physiological influence, but also affect patients’ emotional and social sphere. For these patients who need to face an ostomy after surgery, this is one of the most difficult experiences in their lives. Nevertheless, intervention might be a second chance to keep living in these cases of patients with colorectal cancer, as well as in those cases when that would involve an improvement of symptom control and an increase in the quality of life in people with inflammatory bowel diseases^1^ ^-^ ^2^.

On many occasions, the situation that patients find when they are discharged from hospital is devastating. They do not just have to face the traumatic situation of being aware of a body that has been surgically modified; they also face huge problems when they need specialized care, which might solve their doubts, and need to receive suitable information to adapt them to this new situation. The patient is entitled to receive specialized medical and nursing health care within the preoperative and postoperative period, whether in the hospital or in the Primary Health Care Centre. These patients are likewise entitled to receive counselling before the surgery, in order to ensure that they are fully aware of the benefits of the surgery and the essential facts about coping with a stoma^3^.

In the Spanish context, there are just a few hospitals with stoma care units or units that follow any kind of training protocol for this specialized care before and/or after the surgery. In many cases, patients suffer a lack of suitable information when they are discharged from hospital^4^. Often, the patients themselves have to assume their care^5^.

Few studies have been carried out to explore the perception of patients with a digestive stoma about the health care received. Research has been more focused on quality of life, problems related with stoma and use of devices or the development of complications after the surgical process.

Previous reviews identified non-met needs in these patients, both in Community Care and at home. They addressed key interventions for comprehensive care from inpatient care to transition to home, with special emphasis on care planning and coordination, along with patient education^6^.

In this sense, by promoting self-care, coping mechanisms facilitate patients’ daily life and stress the important role of information and education to empower patients and caregivers in the responsibility for stoma care^7^. A quasi-experimental study based on 110 ostomized patients analyzed the effect of educative interventions provided by nursing. It revealed how the lack of information, communication and education in these patients avoids their participation in self-care. Thus, planned and structured education is a key ingredient for their social rehabilitation^8^.

There is little evidence, however, about strategies to improve health care for ostomized patients from their own experience and point of view^9^. This study is aimed at identifying the perception of people with a digestive stoma about the process of care received and the areas for improvement they detect, as well as their needs and suggestions for this purpose. This is the final study in a research series about the experience of people with a digestive stoma^10^ ^-^ ^12^

## Method

Qualitative study with phenomenological approach. It is based on Husserl’s descriptive phenomenology^13^, as it studies the experience of consciousness as it is, and concludes with a deep analysis that goes beyond the limits of Psychology. The study included patients with GIT stoma, male and female, living in Malaga and Granada (Spain). Criteria for excluding patients were: Patients with cognitive impairment or who rejected to take part in the research. Patients were enrolled by the stoma care nurses working in the University Hospital Virgen de la Victoria in Malaga, University Hospital San Cecilio in Granada and Costa del Sol Hospital in Marbella (Malaga).

Patients’ responses are sensitive to different factors, so the criteria for patient selection were: disease that led to the confirmation of the ostomy (Cancer, Crohn’s disease, ulcerative colitis, familial polyposis); type of intervention (scheduled or urgent surgery); duration of stoma (temporary or permanent) and sociodemographic criteria such as age and gender. The theoretical sampling guidelines were followed^14^, reaching theoretical saturation based on the narratives of 21 patients.

Semistructured interviews were used for data collection, taking 35 to 40 minutes. Interviews took place at the time and place chosen by each patient. All interviews were held face to face by the same researcher and without the presence of other people. The researcher in charge of the interviews held a Master’s degree in Research in Health and Social Sciences at the time of the study. The researcher was not familiar with the subjects who participated in the study. The starting point for the interviews was a guide that included some questions arising from the main issues studied: What did you feel, when you found out you were going to receive a stoma?, What beliefs did you have about the impact the stoma would have in your life?, What feelings did you have when you saw the stoma for the first time?, Until today, how has the creation of the stoma affected you? (In this manuscript, we developed a thematic category derived from the data analysis in relation to the health care received). The interview guide was modified according to the analysis of the first interviews held. These modifications were included as a result of the analysis process, aimed at reaching the theoretical saturation of the data. 

Based on the constant comparison of data and progressive incorporation of new informants, a three-phase sequential scheme was adapted for the data analysis. After reaching the saturation of the data, the analysis was closed off. The phases were: preparation of the data, which included the transcription of the interviews and incorporation in the transcription of the notes from the field notebook collected during and completed after the interviews; organization of the data through the coding of the interviews; and interpretation with detailed reading of the data, constantly comparing the codes and formulating propositions to describe the properties and scope of each category. The analysis was supported by the use of the software Atlas-ti. A triangular three-phase analysis was undertaken in cooperation with other researchers and with stoma therapy nurses who had contacted the informants.

Each participant was previously informed about the nature of the research and his/her participation. It was duly clarified that their participation was voluntary in order to obtain their written or verbal informed consent. They were likewise asked for permission to record the interviews and all the patients included in the research agreed to that. To ensure the anonymity of patients involved in the research, fictitious names were used. The confidentiality of information collected was ensured pursuant to the legislation in force (Law 15/1999, dated December 13^th^). The research received authorization from the Research Committee of the University School of Health Sciences at the University of Malaga.

## Results

The research included the narratives of 21 patients taking part in the research. Twelve of them were men and 9 women, aged from 20 to 75 years. Fourteen patients had a GIT stoma due to an oncological process, 6 of them due to inflammatory bowel diseases and 1 due to familial polyposis. Patients with ileostomy represented 48% and 52% had a colostomy. More than half (62%) had a permanent stoma and 38% a temporary one.

The analysis identified three main categories: Health care received, Health care management and problems met, and needs-suggestions for improvement. 

### Health care received

**Figure 1:**
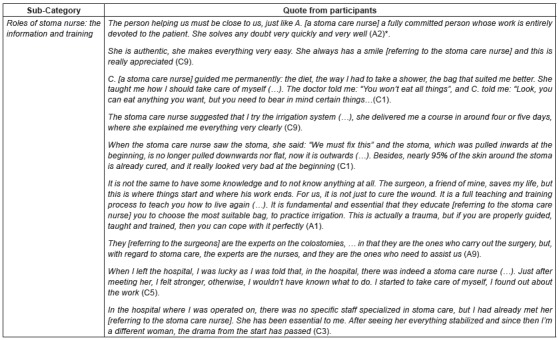
Quotes from category “Health care received”

The functions the informants assign to the stoma care nurse are duly identified, highlighting all those tasks related to the information and training, which enables them to better adapt to their new situation. See quotes of category in Figure 1.

*Alphanumerical codes used to identify participants

Some patients highlighted the importance of trusting somebody who might solve all their doubts and concerns that arise along the process and also encourage them to go ahead.

Another task to be managed by the stoma care nurse is the training to take care of the stoma, the selection of the type of pouching system, diet and information about of the latest advances.

Teaching and the training process about the irrigation and treatment of the problems caused by the stoma and peristomal skin were other functions the patients identified as the responsibility of the stoma nurses. 

One of the issues to be highlighted is the relation between the health care received and the information provided to patients all along the process. All the patients pointed out this factor, as they consider it plays a key role to allow them to face their situation. In this sense, they consider that stoma care nurses are “the experts” on stoma care and that they play a “key role” in their education and information.

Patients also stated that they experienced a change in their lives. It resulted from the influence of a professional who guided them in all the issues concerning the stoma. This fact contributed to get rid of the feeling of uncertainty and fear the unawareness and the lack of information provoked, which promoted their return to their normal life.

With regard to the follow-up process carried out by the stoma care nurse, patients pointed out that they felt calm for knowing they can contact someone who solves their doubts. They also remarked the importance of a follow-up period until they feel self-sufficient.

**Figure 2:**
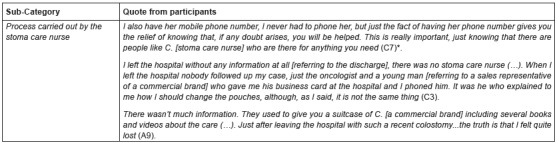
Quotes from category “Health care received”

Relating to patients who had surgery in a hospital without a stoma care nurse service, it was the registered nurse who provided them with information during their hospitalization. Once they were discharged from hospital, however, the manufacturer’s employees assumed that task at best, when they were not confronted with an information void. (Figure 2)

*Alphanumerical code used to identify participants

### Health care management and problems

**Figure 3:**
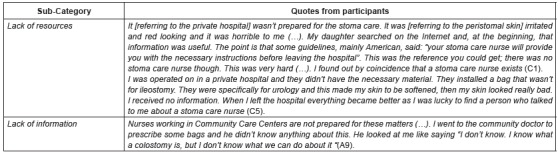
Quotes from category “Health care management and problems found”

Another issue observed about health care management related to the problems patients met regarding the health care services. The patients highlighted complaints about health care management. These complaints are related to medical appointment management, the waiting lists, health care during the holiday period and the lack of resources and trained professional staff to provide health care to patients with GIT stoma. The patients refer to the inconveniences faced, thus involving feelings of uncertainty and desperation. Figure 3 shows quotes from participants.

* Alphanumerical code used to identify participants

The lack of resources and training by the professional staff, jointly with the lack of information received, originated not only feelings of fear, uncertainty and helplessness, but also problems with peristomal skin. It is worth mentioning that these statements came from patients who received health care in private medical centres. It also showed that teams in private medical centres do not offer stoma care nurses.

Lack of information concerning stoma by the professional staff is also identified in Primary Health Care Centres.

### Needs and suggestions for improvement

**Figure 4:**
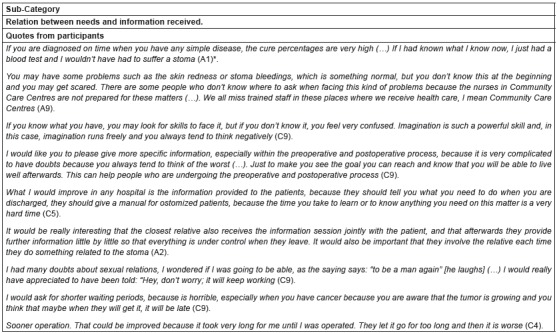
Quotes from category “Needs and suggestions for improvement”

Finally, stoma patients mentioned a number of needs they have to improve and which need to be addressed in the health care system. See quotes from the category in Figure 4.

*Alphanumerical code used to identify participants

The close relation between these needs and the information received should be mentioned.

Some patients request stoma care nurses in all health care stages and highlight the significance of this fact within the scope of the Community Care Centres because that are the places where they usually receive the first health care. Furthermore, patients demand access to information from different types from the very beginning of the process in order to discriminate information to face any situation.

Subsequently, they focus more on the need for preoperative and postoperative information, so as to avoid doubts that might lead them to develop negative feelings. In addition, patients mentioned that the advice they received upon the discharge from hospital should be improved in order to prevent future complications. 

Patients also stressed the importance of family. Their relatives should be properly informed and involved all along the process. One of the needs underlined was to receive more information about sexual relations. Another demand identified is the reduction of the waiting periods, mainly in these cases when time plays against patients, such as oncological diseases.

Finally, some patients with inflammatory bowel diseases mentioned the importance of undergoing the surgery sooner than they did in order to end suffering.

**Figure 5:**
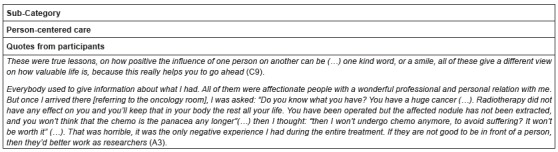
Quotes from category “Needs and suggestions for improvement”

Person-centered care is claimed to be an useful tool that helps them to go ahead. Patients also mentioned that nursing professionalism plays an important role within the process. It makes reference to the idea that, if you work with people, you need to be ready for such a task (Figure 5).

*Alphanumerical code used to identify participants

## Discussion

This study was aimed at obtaining information about the perception of people with a digestive stoma regarding the process of care received and the areas for improvement they detected, as well as their needs and suggestions for improvement.

One of the main findings was that information is one of the most relevant issues defining the relation established between ostomy patients and health care providers. Both those patients who received suitable information and those who did not do highlight the importance of such information within the health care process in order to face the situation and get back to normal life.

Patients pointed out tasks performed by stoma care nurses, as they are considered to be the main person of reference within the health care process. Thus, they believe that the health care system should be more focused on training nurses in stoma care and should contract them. Different studies confirm that management care done by nurses trained in stoma has a positive impact in patients during the process of adaptation to the stoma^15^ ^-^ ^17^. 

It is worth mentioning the close relation between the skills and level of specialisation and the care provided by nurses, as well as the importance of engaging other people in the process, as other authors also pointed out^4^.

Information is a basic resource to develop coping strategies and plays a predominant role throughout the process, as stated in different studies^17^ ^-^ ^19^. That information should be provided not only to patients, but also to the closest relatives, who need to be more involved in the process.

Needs and improvements patients suggested during the interviews appoint that the patient is entitled to receive specialized medical and nursing care within the preoperative and postoperative period, not just in the hospital but also in Primary Care. These patients are likewise entitled to receive counselling before the surgery in order to ensure they are fully aware of the benefits of the operation and receive suitable information on the essential facts about living with a stoma^3^.

Within our scope, however, these rights are not always guaranteed and this fact leads the stoma patient to feel helplessness on some occasions after they are discharged from hospital^17^ ^-^ ^18^ ^,^ ^20^ ^-^ ^21^. They also need to train on their own and develop information seeking behaviours until they find specialized staff^3^.

Additionally, stoma patients identified further needs that are also important, although not directly related with the lack of information, such as: the reduction of the waiting periods or the importance of being operated on in earlier stages of the disease in order to end their suffering. Without this, they will suffer uncertainty and desperation. These patients who underwent the process during a holiday, such as the summer or Christmas period, mentioned that they missed information in both the preoperative and postoperative period, as well as training offered by professional staff, entailing a lack of confidence for many of them.

*Limits of the study.* With regard to the limitations of this research, the patients who took part were mainly patients whose disease that caused the stoma was an oncological process. Despite the representativeness of this group, we consider it is appropriate to study individuals with a GIT stoma whose experiences with the disease are different. According to the outcome of the research, the importance the patients attribute to their own autonomy is noted. Nevertheless, their statements do not make clear when they became aware of their autonomy or the reasons that led them to this awareness. Therefore, some questions remain about the promoting periods that help to develop personal autonomy within the self-care of the disease and the circumstances that favour it. The transferability to other contexts requires further study.

Although people with temporary stoma were included in this study, there are evidences that show that people with temporal stoma may influence patients’ adaptation. Nevertheless, strategies for this type of patients may be developed within the same context, as they need more specific attention. 

## Conclusions

The perception of the health care received is closely linked to the information and communication process experienced. Regardless of the nature of the information received, this is considered to play a key role in order to face the situation and get back to normality. It also affects the quality of life. The importance of the stoma care nurse in all stages of the health care is specifically stressed, being the professional of reference to obtain support. 

Patients pointed out important needs that were not met as a result of a slightly rationalized healthcare service that is very variable with regard to service access, waiting periods, specific training of the professional staff in information and health care coordination. Some of these needs informed should be included in the design and development of services for Ostomized patients, or in the redesign of the current health care provided to them.
